# Drivers of Bacterial α- and β-Diversity Patterns and Functioning in Subsurface Hadal Sediments

**DOI:** 10.3389/fmicb.2019.02609

**Published:** 2019-11-14

**Authors:** Eugenio Rastelli, Cinzia Corinaldesi, Antonio Dell’Anno, Michael Tangherlini, Marco Lo Martire, Manabu Nishizawa, Hidetaka Nomaki, Takuro Nunoura, Roberto Danovaro

**Affiliations:** ^1^Department of Marine Biotechnology, Stazione Zoologica Anton Dohrn, Naples, Italy; ^2^Department of Materials, Environmental Sciences and Urban Planning, Polytechnic University of Marche, Ancona, Italy; ^3^Department of Life and Environmental Sciences, Polytechnic University of Marche, Ancona, Italy; ^4^Department of Research Infrastructures for Marine Biological Resources, Stazione Zoologica Anton Dohrn, Naples, Italy; ^5^Institute for Extra-cutting-edge Science and Technology Avant-garde Research (X-star), Japan Agency for Marine-Earth Science and Technology (JAMSTEC), Yokosuka, Japan; ^6^Research Center for Bioscience and Nanoscience (CeBN), Japan Agency for Marine-Earth Science and Technology (JAMSTEC), Yokosuka, Japan

**Keywords:** hadal trench biosphere, subsurface sediments, bacteria, viruses, sediment geochemistry, bacterial biodiversity, organic matter cycling

## Abstract

Oceanic trenches at hadal (>6,000 m) depths are hot spots of organic matter deposition and mineralization and can host abundant and active bacterial assemblages. However, the factors able to shape their biodiversity and functioning remain largely unexplored, especially in subsurface sediments. Here, we investigated the patterns and drivers of benthic bacterial α- and β-diversity (i.e., OTU richness and turnover diversity) along the vertical profile down to 1.5 m sediment depth in the Izu-Bonin Trench (at ~10,000 m water depth). The protease and glucosidase enzymatic activity rates were also determined, as a proxy of organic matter degradation potential in the different sediment layers. Molecular fingerprinting based on automated ribosomal intergenic spacer analysis (ARISA) indicated that the α-diversity of bacterial assemblages remained high throughout the vertical profile and that the turnover (β-) diversity among sediment horizons reached values up to 90% of dissimilarity. Multivariate distance-based linear modeling (DISTLM) pointed out that the diversity and functioning of the hadal bacterial assemblages were influenced by the variability of environmental conditions (including the availability of organic resources and electron donors/acceptors) and of viral production rates along the sediment vertical profile. Based on our results, we can argue that the heterogeneity of physical-chemical features of the hadal sediments of the Izu-Bonin Trench contribute to increase the niches availability for different bacterial taxa, while viruses contribute to maintain high levels of bacterial turnover diversity and to enhance organic matter cycling in these extremely remote and isolated ecosystems.

## Introduction

The hadal biosphere is almost exclusively represented by ultra-abyssal trenches and comprises some of the most remote and inaccessible deep-sea environments, whose microbial ecology is still largely unexplored (Jamieson, 2015, 2018; Jamieson et al., 2018). Deep-sea benthic bacterial assemblages play a major role in biogeochemical cycles, food webs, and overall marine ecosystems’ functioning (Lozupone and Knight, 2007; Mason et al., 2009; Zinger et al., 2011; Danovaro et al., 2014, 2015; Corinaldesi, 2015; Lloyd et al., 2018), yet the knowledge on their biodiversity and functioning in hadal trenches is still very scant and thus a timely and challenging frontier in microbiology (Jamieson et al., 2010; Nunoura et al., 2015). Moreover, the hadal subsurface biosphere remains even more understudied, as the current knowledge on the distribution, diversity, and metabolic potential of bacterial assemblages in benthic hadal ecosystems and on their environmental drivers mainly relies on few surficial sediment samples (Kato et al., 1997; Li et al., 1999, 2019; Nunoura et al., 2013, 2018; Yoshida et al., 2013; Wenzhöfer et al., 2016; Jamieson et al., 2018; Cui et al., 2019; Peoples et al., 2019).

Independent evidence suggests that the enhanced deposition of sinking and resuspended particles to the bottom due to the peculiar “V”-shaped trench topography (Wenzhöfer et al., 2016; Xu et al., 2018) can support more abundant and metabolically active bacterial assemblages than in the abyssal surroundings (Danovaro et al., 2003; Glud et al., 2013; Leduc et al., 2016; Wenzhöfer et al., 2016). This suggests that hadal bacteria interact with and are influenced by changes in the trench sedimentation regime.

The stimulation of benthic heterotrophic metabolism at the hadal seafloor leads to higher rates of oxygen consumption in the sediments and results in steeper geochemical gradients along the vertical profile of hadal sediments compared with typical open-ocean abyssal sites (Glud et al., 2013; Nunoura et al., 2013; Wenzhöfer et al., 2016). This feature has been suggested to contribute to influence the composition of bacterial assemblages down the hadal sediment profile (Nunoura et al., 2013, 2018; Li et al., 2019: Peoples et al., 2019).

In addition, the distribution, diversity, and functioning of the hadal bacterial assemblages could be influenced by the episodic and scattered deposition of heterogeneous materials enhanced by frequent earthquakes, volcanic eruptions, and turbidite flows, which contribute to diversify the seafloor features in these tectonically active zones (Nozaki and Ohta, 1993; Oguri et al., 2013; Leduc et al., 2016; Gamo and Shitashima, 2018; Ikehara et al., 2018; Kawagucci et al., 2018; Luo et al., 2018).

Among the biotic factors able to shape the diversity and functioning of bacterial assemblages, viruses are expected to play a more relevant role in deep-sea and subsurface sediments due to the concomitantly reduced grazing by eukaryotes (Danovaro et al., 2008; Engelhardt et al., 2014, 2015). Indeed, recent evidences indicate that the rates of viral infections in hadal sediments can be as high as in other deep-sea environments and that the impact of viruses on archaea can be particularly relevant (Danovaro et al., 2016). However, the current knowledge on the effects of viruses on the diversity and functioning of hadal bacterial assemblages remains largely unexplored, especially for subsurface hadal sediments (Glud et al., 2013; Yoshida et al., 2013; Danovaro et al., 2016; Nunoura et al., 2018).

In this study, we collected sediment samples down to 1.5 m depth below the seafloor, at ca. 10,000 m depth in the largest benthic hadal habitat on Earth (the Izu-Bonin Trench; Stewart and Jamieson, 2018), to investigate α- and turnover (β-)diversity of bacterial assemblages, their functioning and abiotic and biotic drivers (organic resources, sediment geochemistry, and viral infections).

## Materials and Methods

### Sediment Sampling and Sample Processing

**Figure 1:**
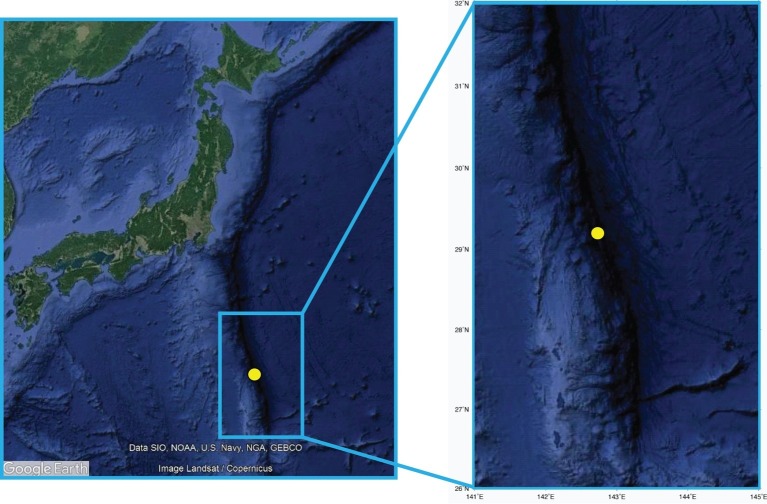
The sampling site in the Izu-Bonin Trench. The maps report the location of the site investigated in the present study, with the enlarged highlight showing high-resolution bathymetry details.

Sediment samples were collected in December 2011 using the ROV ABISMO gravity core (Yoshida et al., 2009) in one of the deepest parts of the Izu-Bonin Trench (29°09′N, 142°48′E) at 9,776 m depth (Figure 1). This site was selected for consistency with previous cruises in the same area in order to make appropriate comparisons (Nunoura et al., 2013; Yoshida et al., 2013). One 1.55-m long sediment core was retrieved during the Japan Agency for Marine-Earth Science and Technology (JAMSTEC) *R/V Kairei* KR11-11 cruise. Oxygen concentrations were measured on the intact core immediately after core recovery by inserting oxygen microelectrodes with stainless guard needle (Unisense, Denmark), calibrated with oxygen-saturated and oxygen-free seawater, through holes in the core opened at every 2–10 cm intervals. The sediment core was then sliced in different layers using sterilized top-cut 50-ml syringes or spatulas. Subaliquots of the sediment intervals 0–1, 10–15, 15–20, 30–35, 45–55, 55–65, 95–105, 115–125, and 145–155 cm were used to carry out the analysis of the organic matter composition and microbiological parameters. Immediately after sediment collection, the samples for the analyses of the organic matter composition were stored at −20°C. For the analyses of prokaryotic abundances, samples were fixed with sterile 0.2-μm-pre-filtered formaldehyde solution onboard and stored at −80°C until further processing on land. For the molecular analyses, sediment subsamples were immediately stored at −80°C until further processing. All frozen samples were processed within 4 months after sediment collection. Sediment pore waters were extracted by centrifuging at 2,600 × *g* for 5–10 min, then filtrated through a 0.45-μm membrane filter. Nitrate and ammonium ion concentrations were measured spectrophotometrically using an automated analyzer QuAAtro2-HR (BL TEC, Osaka, Japan). pH was measured by a compact pH meter LAQUAtwin (Horiba, Kyoto, Japan). The analyses of viral production and extracellular enzymatic activity (β-glucosidase and aminopeptidase) were conducted immediately after the collection of sediments by means of time course experiments at *in situ* temperature in the dark, as described in the following sections. All variables were analyzed in at least three replicates, and all data were normalized to sediment dry weight after desiccation (48 h at 60°C).

### Organic Matter Composition and Turnover

Total organic carbon (TOC) and total nitrogen (TN) were measured with an elemental analyzer (Flash EA1112-DELTA V ADVANTAGE ConFloIV System, Thermo Fisher Scientific). Before the measurements, the sediment samples were freeze-dried and acidified with 1 M HCl to remove inorganic carbon. The decalcified sediments were then dried, weighed, and put into pre-cleaned tin cups.

The concentration of proteins, carbohydrates, and lipids in the collected sediments was measured using photometric protocols (Danovaro, 2010). The sum of the carbohydrate, protein, and lipid concentrations converted into carbon equivalents (by using the conversion factors of 0.40, 0.49, and 0.75 μgC μg^−1^, respectively) was defined as biopolymeric carbon (BPC, Danovaro, 2010). The turnover times of proteins and carbohydrates in the sediment, used as a proxy of protein and carbohydrate cycling efficiency, were calculated as the ratio of the total protein or total carbohydrate concentrations and their degradation rates determined based on aminopeptidase and β-glucosidase enzymatic activity rates. These degradation rates were determined by the analysis of the cleavage rates of the artificial fluorogenic substrates L-leucine-4-methylcoumarinyl-7-amide (Leu-MCA) and 4-methylumbelliferyl (MUF)-b-D-glucopyranoside (Glu-MUF) (Sigma Chemicals, Milan, Italy) for aminopeptidase and β-glucosidase, respectively, under saturating substrate concentrations (Danovaro, 2010). Briefly, sediment subsamples were diluted with 0.02-mm pre-filtered seawater collected at the water-sediment interface, and incubated in the dark at the *in situ* temperature for 1 h. The fluorescence of the samples was measured fluorometrically at 380 nm excitation, 440 nm emission (for Leu-MCA) and 365 nm excitation, 455 nm emission (for Glu-MUF), immediately after the addition of the substrate and after the incubation. The fluorescence was then converted into enzymatic activity based on standard curves obtained using standard 7-amino-4-methylcoumarin (Sigma Chemicals) for Leu-MCA and 4-methylumbelliferone (Sigma Chemicals) for Glu-MUF. The cell-specific degradation rates were calculated by the ratio between enzymatic activity and cell abundance. The amount of the artificial fluorogenic substrate hydrolyzed by proteases and glucosidases were converted respectively into protein and carbohydrate degradation rates using 72 mg of C per micromole of substrate hydrolyzed. The organic matter turnover was expressed as the ratio of the enzymatic C degradation rates and the protein and carbohydrate C contents in the sediment (Pusceddu et al., 2014).

### Epifluorescence Microscopy Analyses on Bacteria, Total Prokaryotic Cells, and Viruses

A standard catalyzed reporter deposition fluorescence *in situ* hybridization (CARD-FISH) was applied for the quantification of bacteria as described in detail in the previous work (Pernthaler et al., 2002; Danovaro et al., 2016). Briefly, sediment samples were washed with phosphate-buffered saline (PBS) (145 mM NaCl, 1.4 mM NaH_2_PO_4_, 8 mM Na_2_HPO_4_; pH 7.6), centrifuged, and suspended in 1:1 solution of PBS: 96% ethanol. After sonication (Branson Sonifier 2,200, 60 W; three sonication cycles of 1 min each) samples were diluted, filtered onto 0.2-μm filters, and dipped in low-gelling point agarose [0.1% (wt/vol) in Milli-Q water], dried on Petri dish at 37°C, and dehydrated in 95% ethanol. Bacterial cell wall permeabilization was achieved by incubation at 37°C with lysozyme (Sigma Chemicals). After Milli-Q water washing and incubation in 10 mM HCl (room temperature, 20 min), the filters were washed again, dehydrated in 95% ethanol, dried, and hybridized with oligonucleotide horseradish peroxidase (HRP)-labeled probes Eub-mix (Eub338, Eub338-II, and Eub338-III) targeting bacteria, while the absence of nonspecific signals was routinely checked using the NON-338 probe (Pernthaler et al., 2002). Hybridization was performed at 35°C for 2 h. Then, the filters were transferred into preheated washing buffer, placed in PBS buffer (pH 7.6, 0.05% Triton X-100), and incubated at room temperature for 15 min. After removal of the buffer, the samples were incubated for 30 min in the dark at 37°C for Cy3-tyramide signal amplification. Filters were analyzed using epifluorescence microscopy (Zeiss Axioskop 2MOT, Jena, Germany) (magnification × 1,000). For each filter, at least 20 microscope fields were observed under green light and at least 400 cells were counted. The total prokaryotic abundance was assessed by staining filters with 20 μl of SYBR Green I (10,000× stock) diluted 1:20 in pre-filtered TE buffer (10 mM Tris-HCl, 1 mM EDTA, pH 7.5) (Danovaro, 2010). Excess stain was removed three times using 3 ml of Milli-Q water, then filters were mounted on microscope slides and counted under blue light, as described above.

Viral abundance was determined by epifluorescence microscopy after extraction of viruses from the sediment using pyrophosphate (final concentration, 5 mM), sonication (Branson Sonifier 2200, 60 W; three sonication cycles of 1 min each), and staining with SYBR Green I (Danovaro, 2010; Rastelli et al., 2016). Viral production was determined by time-course experiments of sediment samples previously diluted 1:10 vol:vol with virus-free seawater (0.02 μm pre-filtered) collected at the water-sediment interface (Dell’Anno et al., 2009; Rastelli et al., 2016). Replicate samples (*n* = 3) for viral counts were collected immediately after dilution of the sediments and after 3, 6, and 12 h of incubation at *in situ* temperature in the dark. At selected subsurface sediment layers [33 and 150 cm below the seafloor (cmbsf)], parallel aliquots were incubated as undiluted samples with no addition of oxygen-containing seawater and no homogenization to exclude possible biases of sediment oxygenation and manipulation on viral production rates (Rastelli et al., 2016). Sediment subsamples were then analyzed as reported for the determination of viral abundance, and viral production rates were determined from linear regression analyses of the increase of viral abundances over time (Dell’Anno et al., 2009; Rastelli et al., 2016). To estimate the rates of carbon released by viral lysis of bacterial cells and the virus-induced turnover of bacteria, we assumed values obtained previously for the same sediments (i.e., 65% of the total viral production due to bacteria; average burst size of 35 viruses per lysed cell; bacterial biomass of 46 fgC cell^−1^) (Danovaro et al., 2016).

### Molecular Analyses

The microbial DNA (i.e., intracellular DNA) was recovered from the sediment samples following specific procedures previously developed (Corinaldesi et al., 2005). Briefly, the sediment samples were treated with acid-washed polyvinylpolypyrrolidone (0.05% final concentration) and sodium dodecyl sulfate (final concentration, 0.1%). Then the samples were chilled on ice, centrifuged, and the supernatants were transferred to sterile tubes. The sediment pellets were washed again six times with sodium phosphate buffer (pH 8.0), then the supernatants were combined and centrifuged for 20 min at 10,000 × *g* (4°C). The resulting pellets containing prokaryotic cells were treated with DNase I to exclude any possible contamination from residual extracellular DNA (Corinaldesi et al., 2005) and then processed for DNA extraction by using the DNeasy PowerSoil Kit (Qiagen, Milan, Italy) according to the manufacturer’s instructions. All DNA extractions were conducted in duplicates. The purified extracts of intracellular DNA were then analyzed by molecular fingerprinting (through ARISA—Automated Ribosomal Intergenic Spacer Analysis) (Hewson and Fuhrman, 2004) to provide information on the bacterial diversity in the analyzed sediment samples. For ARISA, the purified intracellular DNA was amplified using universal bacterial primers 16S-1392F (5′-GYACACACCGCCCGT-3′) and 23S-125R (5′-GGGTTBCCCCATTCRG-3′) (Danovaro et al., 2006). This allowed the amplification of the ITS1 region in the rRNA operon plus ~282 bases of the 16 S and 23 S rRNA72. Primer 23S-125R was fluorescently labeled with the fluorochrome HEX (Eurofins, Milan, Italy). PCR reactions were performed in 50 μl volumes in a thermalcycler (Biometra, Göttingen, Germany) using the MasterTaq® kit (Eppendorf, Milan, Italy). The amplicons were obtained using 30 PCR-cycles, consisting of 94°C for 1 min, 55°C for 1 min, and 72°C for 2 min, preceded by 3 min of denaturation at 94°C. To check for eventual contamination of the consumables and reagents, negative controls of DNeasy PowerSoil Kit extracts from blank samples (i.e., control extractions with no sediment sample) containing the PCR-reaction mixture were run during each PCR reaction. All the negative controls produced no ARISA amplicons, confirming the absence of contamination and the high confidence of the analytical approach. Positive controls containing genomic DNA of *E. coli* were used, and the PCR amplicons were confirmed on agarose-TBE gel (1%), containing GelRed^™^ DNA staining (Biotium, Fremont, USA) for UV visualization. For each sample, eight PCR reactions (i.e., four reactions for each of the two replicated DNA extracts) were run and then combined to form four replicates for independent ARISA reactions. The PCR products were purified using the Wizard PCR clean-up system (Promega, Milan, Italy) and resuspended in 50 μl of sterile MilliQ water. For each ARISA run, 5 ng of purified amplicons were mixed with 14 μl of internal size standard (GS2500-ROX; Applied Biosystems, Foster City, USA) in deionized formamide, then denatured at 94°C for 2 min and immediately chilled in ice. Automated detection of ARISA fragments were carried out using ABI Prism 3100 Genetic Analyzer. ARISA fragments were determined using Peakscanner analytical software (Applied Biosystems) and the results analyzed using standardization of fluorescence among samples, elimination of “shoulder” and non-replicated peaks, and cut-off criterion (Danovaro et al., 2006). The bacterial α-diversity was expressed as the total number of peaks within each electropherogram (i.e., the cumulative number of bacterial OTUs identified by ARISA in each sample), while the evenness (Pielou index, *J*’) was calculated in order to assess the relative importance of each bacterial OTU within the entire assemblage. We then calculated bacterial turnover (β-)diversity as percentage of Bray-Curtis dissimilarity among samples using the SIMPER tool of the PRIMER 6+ software (Plymouth Marine Laboratory, Plymouth, UK) (Clarke and Gorley, 2006; Luna et al., 2013). Values of turnover (β-)diversity were calculated considering all possible combinations in the comparison of the bacterial OTU profiles between different sediment layers (i.e., four replicates per sediment layer, pairwise-compared with those of the other eight sediment layers, for a total of 32 possible comparisons for each sediment layer).

### Statistical Analyses

Differences in the investigated variables (organic matter concentrations, geochemical parameters, bacterial and viral abundances, viral production rates, enzymatic activities, and α-/β-diversity and evenness) between different sediment layers were assessed through the analysis of variance (ANOVA). Data of bacterial assemblage composition obtained from the ARISA profiles were analyzed using multivariate statistical tools to test the hypothesis that differences exist in bacterial assemblage composition among different sediment layers, and data were ordinated by clustering analysis based on Bray-Curtis similarity. To identify the abiotic and biotic drivers (ammonium, nitrate, and oxygen concentrations; protein-to-carbohydrate ratio; biopolymeric carbon, and total carbon concentration; and viral production) able to influence, bacterial α- and β-diversity and organic matter turnover, multivariate multiple regression analyses were carried out, using distance-based multivariate analysis for a linear model (DISTLM) with the forward routine (Anderson, 2004). *p*’s were obtained using 4,999 permutations of residuals under a reduced model. All statistical analyses were performed using the PRIMER 6+ software (Anderson et al., 2008).

## Results and Discussion

### Steep Vertical Gradients in the Biogeochemical Properties of Hadal Sediments

**Figure 2:**
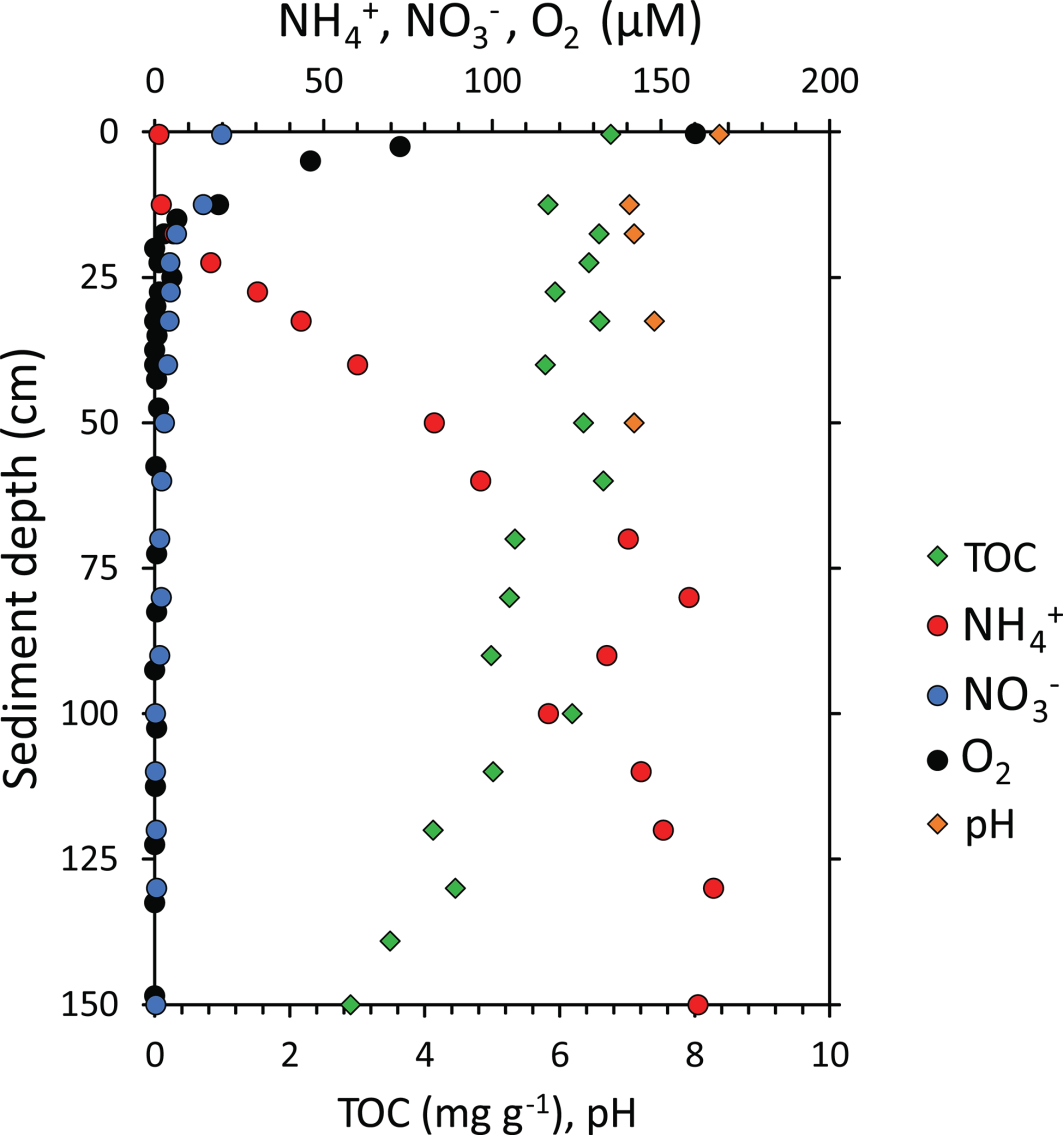
Geochemical characterization of the sediment core collected from the Izu-Bonin Trench. Reported are the concentrations of total organic carbon (TOC), ammonium (${NH}_{4}^{+}$), nitrate (${\mathrm{NO}}_{3}^{-}$), and oxygen (O_2_) concentrations and pH along the vertical profile of hadal sediments.

In the present study, we found clear differences and steep gradients in the geochemistry among the sediment layers of the 1.55-m-long sediment core collected from one of the deepest points of the Izu-Bonin Trench in the Pacific Ocean (Figure 2). Oxygen concentration decreased sharply from 160 μM at the sediment surface to >1 μM below 20 cmbsf, indicating fast oxygen consumption by the benthic community within the surface layers of the hadal sediments. This trend is confirmed by independent *in situ* measurements conducted in the same trench at similar water depth (Wenzhöfer et al., 2016). Ammonium concentrations were <6 μM above 20 cmbsf of the sediments and increased up to 160μM in the deeper sediment layers, while nitrate concentrations decreased from 20μM at the sediment surface to <5μM at 30 cmbsf. Such profiles for oxygen, ammonium and nitrate concentrations support previous hypotheses dealing with the coexistence of aerobic and anaerobic microbial metabolic processes along the sediment profile in the Izu-Bonin Trench (Nunoura et al., 2013). Such a pattern, observed also in other trenches (Glud et al., 2013; Wenzhöfer et al., 2016), is common in organic-rich sediments, and it differs markedly with that of oligotrophic abyssal sediments of the Pacific Ocean, in which oxygen and/or nitrate respiration persists down to >2 m sediment depth (Durbin and Teske, 2011).

**Figure 3:**
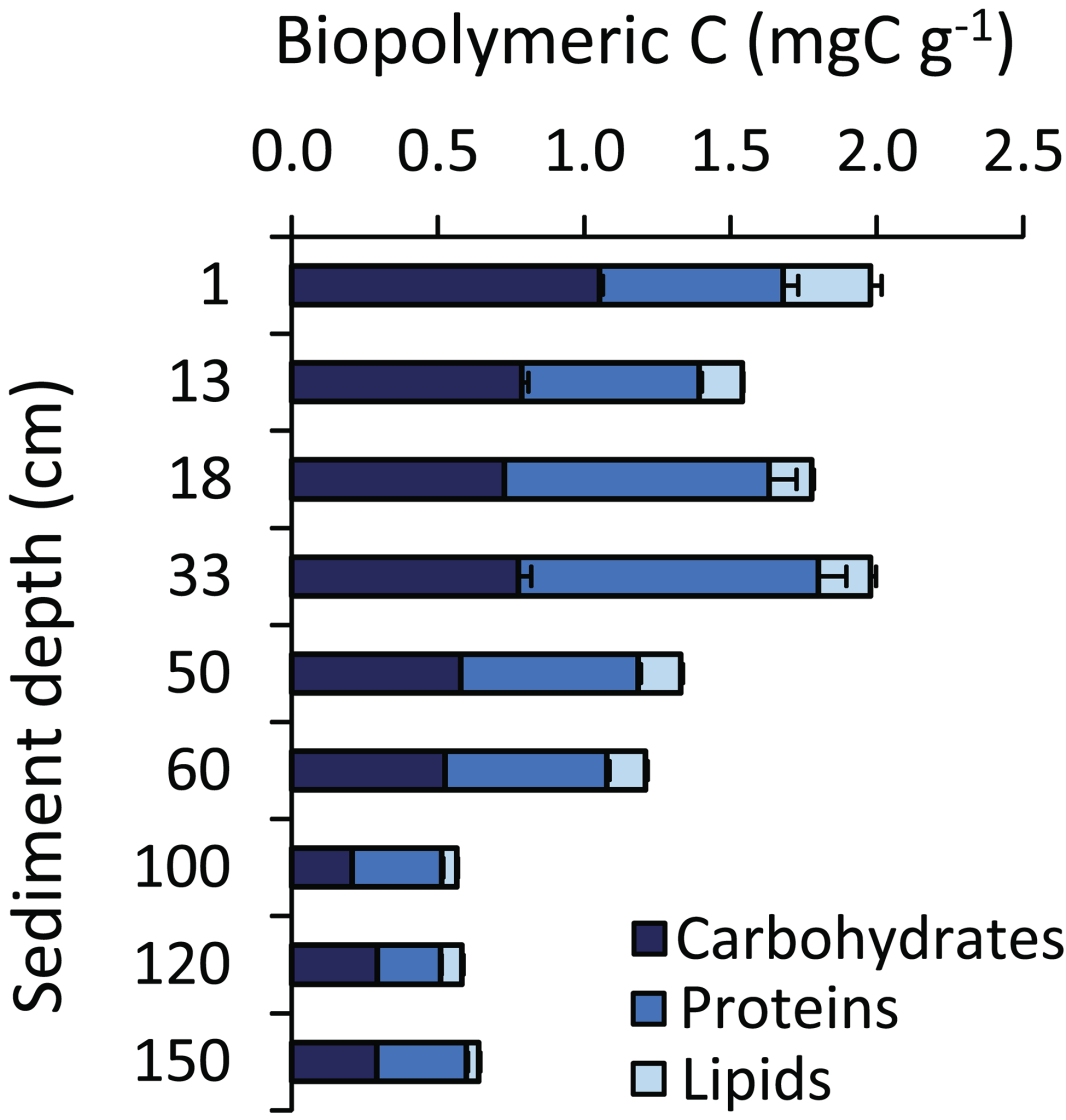
Biochemical composition of the sedimentary organic matter. The figure shows, for each hadal sediment layer, the cumulative value of the carbon content of the different fractions of the biopolymeric carbon (i.e., carbohydrates, proteins, and lipids). Reported are average values and SDs.

The heterogeneity in the geochemical conditions along the sediment depth profile was evident also in terms of pH, total organic carbon (TOC), and biopolymeric carbon (BPC) concentrations (Figures 2, 3). Sediment pH displayed higher values (8.4) in well-oxygenated surface sediments, decreasing to approx. 7.0–7.4 in subsurface sediments. According to the results obtained by Wenzhöfer et al. (2016), TOC concentrations were quite constant in the top 60 cmbsf sediments with highest value of 6.8 mg g^−1^ in the top layer, then showing a two-fold decrease down to <3 mg g^−1^ at the bottom of the sediment core (Figure 2). BPC concentrations decreased four-fold along the sediment profile (from 2.1 to 0.6 mg g^−1^; Figure 3) suggesting not only a lower quantity but also a lower quality of the organic resources in the deeper sediment layers (i.e., a reduced content of labile organic compounds available for heterotrophic consumers; Danovaro et al., 2014).

### Hadal Subsurface Sediments Host High Bacterial and Viral Abundances and Viral Production Rates

**Figure 4:**
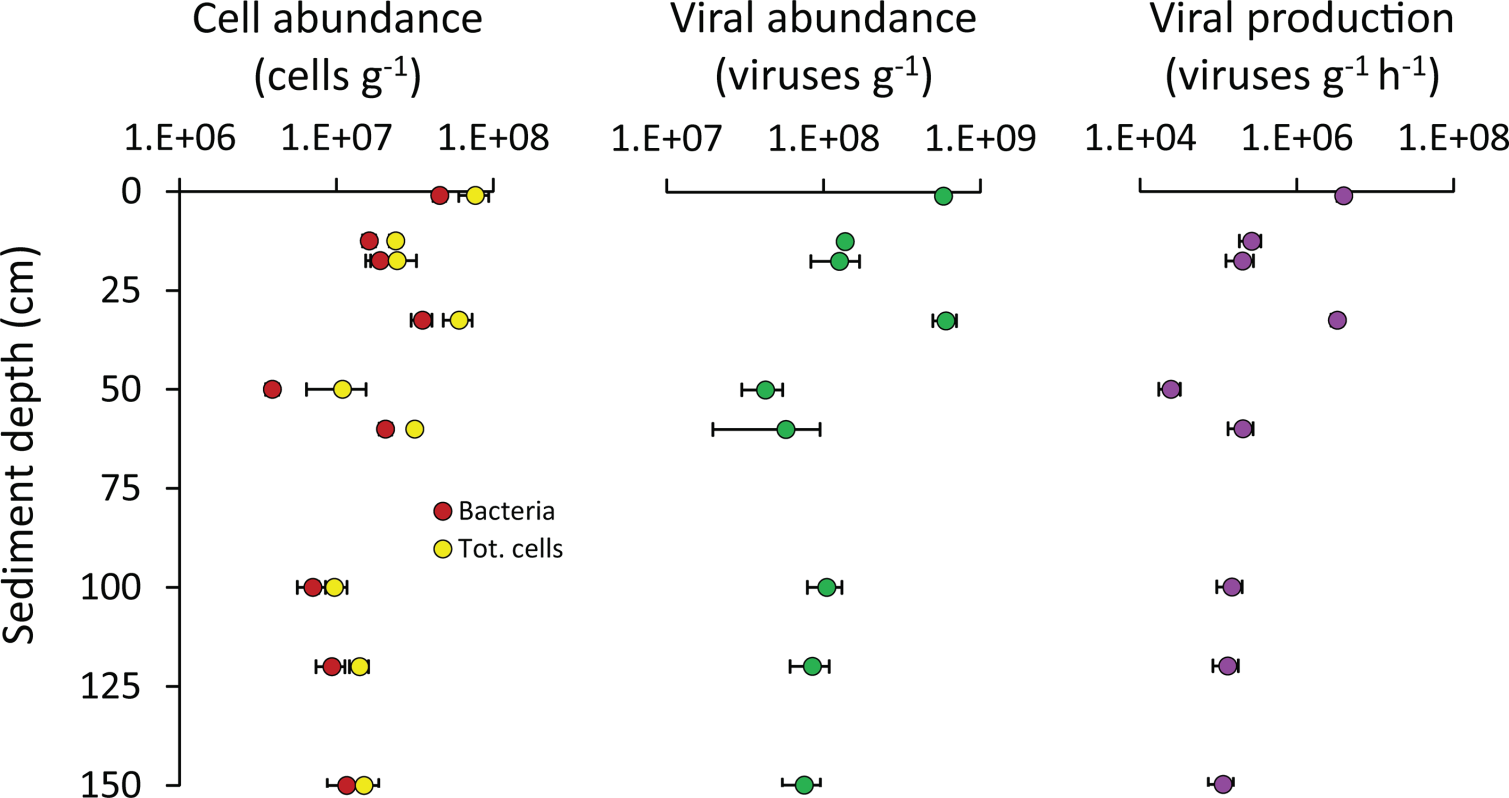
Cell abundance, viral abundance, and viral production. Reported are average values and SDs for the abundances of bacteria and of total prokaryotic cells, as well as for the viral abundance and production rates, determined along the vertical profile of the hadal sediments analyzed in the present study.

The bacterial abundances in the Izu-Bonin Trench sediments ranged from 3.5 × 10^6^ cells g^−1^ at 50 cmbsf to 5.0 × 10^7^ cells g^−1^ in the top layer (Figure 4). These values fall in the range of those observed in abyssal sediments of other oceanographic areas, including the Atlantic and Arctic Ocean and the Mediterranean Sea (Danovaro et al., 2015, 2016). Overall, these abundances showed an unclear pattern along the vertical profile of the sediments, with values in the layers deeper than 50 cmbsf comparable to those of the upper layers (Figure 4). This trend also characterized the total prokaryotic abundances, which ranged from 6.4 × 10^6^ cells g^−1^ at 50 cmbs to 9.4 × 10^7^ cells g^−1^ in the top layer, confirming the range of values reported in previous independent investigations in this area (Wenzhöfer et al., 2016). The relatively low contribution of the top sediment layer to the total (integrated) cell abundance along the sediment vertical profile is consistent with evidences obtained for abyssal sediments (Whitman et al., 1998; Kallmeyer et al., 2012; Danovaro et al., 2015), indicating that also in hadal trenches most of the total cell standing stock resides in the subsurface sediments.

Besides biogeochemical parameters, viruses also are known to profoundly influence the microbial assemblages at the deep seafloor (Danovaro et al., 2008; Engelhardt et al., 2014, 2015; Orsi, 2018), thus possibly contributing to shape the observed patterns of diversity and functioning of the benthic bacterial assemblages of the Izu-Bonin Trench. The abundance of viruses in the Izu-Bonin Trench sediments ranged from 3.0 × 10^7^ viruses g^−1^ at 50 cmbs to 5.9 × 10^8^ viruses g^−1^ in the top layer, while the viral production rates ranged from 1.7 × 10^4^ viruses g^−1^ h^−1^ at 50 cmbs to 4.7 × 10^6^ viruses g^−1^ h^−1^ in the top layer. In the subsurface sediments, viral abundances reached values up to 7.1× 10^8^ viruses g^−1^ and production rates of 4.0 × 10^6^ viruses g^−1^ h^−1^ (in the 33-cmbsf layer; Figure 4). Such values were similar or even higher than in the top sediment layer (Figure 4) and compared with values reported for abyssal surface sediments (Danovaro et al., 2008, 2016) and for other hadal sediments (Yoshida et al., 2013). The turnover of viruses was faster in the surface sediments and in the 33-cmbsf layer (6.9 ± 1.0 days) than in the other subsurface layers (31 ± 7 days). This is consistent with an expected decrease of microbial metabolic rates (and hence, of viral replication) down the sediment profile (Hoehler and Jørgensen, 2013; Orsi, 2018), at the same time suggesting that some hadal subsurface sediment layers might host particularly active virus-host interactions at rates comparable to those in surface sediments.

Bacterial abundances positively correlated with both viral abundances (*y* = 14.7; *x* = −7.5 × 10^7^; *R*^2^ = 0.820) and viral production (*y* = 0.10; *x* = −9.9 × 10^5^; *R*^2^ = 0.823; Supplementary Figure S2), suggesting that larger bacterial standing stocks support higher viral abundances and production rates in subsuperficial hadal sediments.

Values of viral production provided here, obtained using the dilution technique (Dell’Anno et al., 2009; Rastelli et al., 2016) represent a reliable estimate of the viral replication rates as the incubations conducted in parallel on undiluted sediments provided values not significantly different (Supplementary Figure S1). Previous works have postulated an active role of viruses in the subsurface biosphere based on indirect evidences such as virus-to-prokaryote ratios and/or genetic information (Engelhardt et al., 2014, 2015; Labonté et al., 2015; Parikka et al., 2017). Thus, our results provide novel and independent evidence of the high replication of viruses also in the hadal subsurface sediments of the Izu-Bonin Trench.

### High α- and β-Diversity of Benthic Bacterial Assemblages in the Izu-Bonin Trench

**Figure 5:**
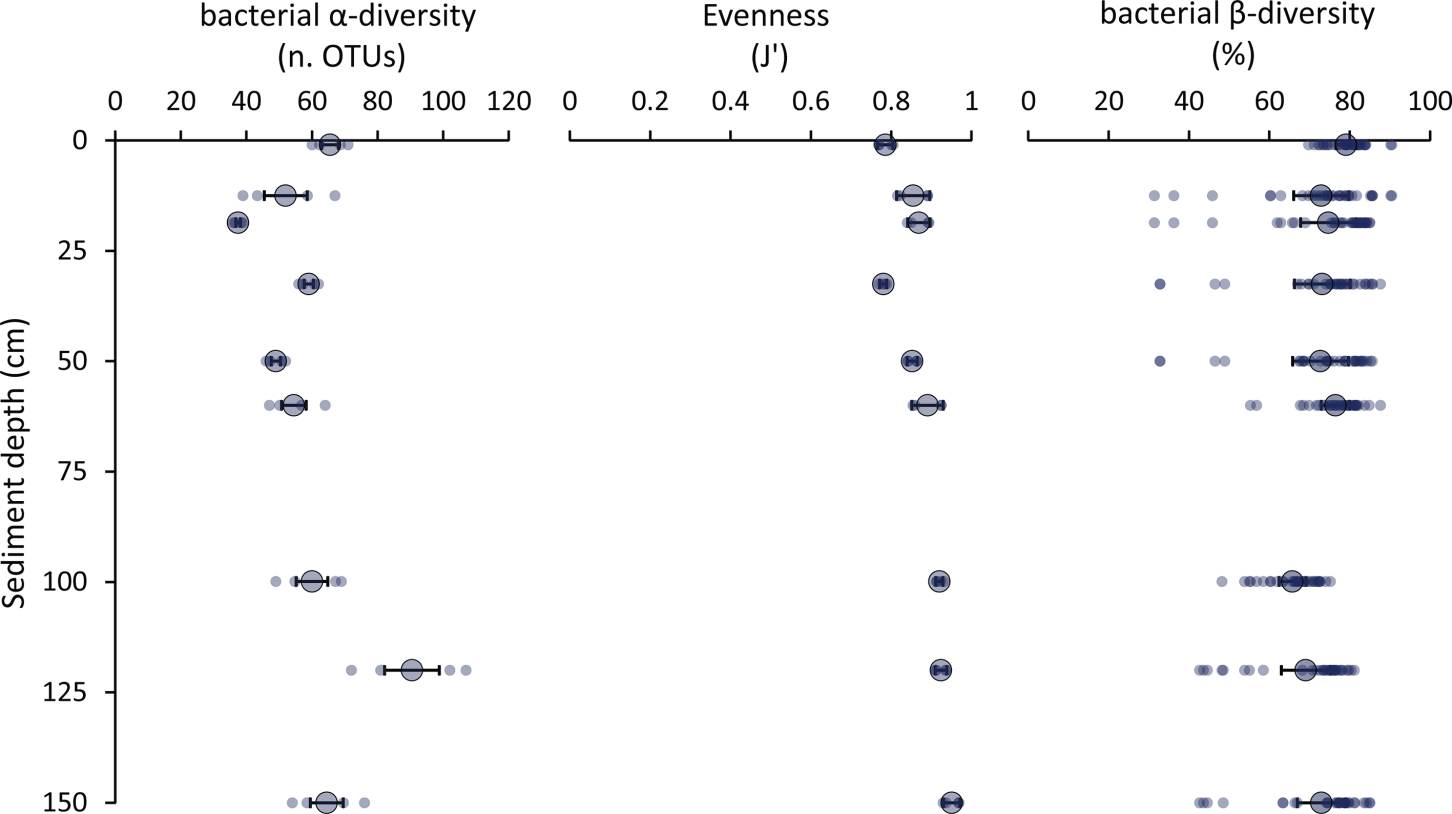
Patterns of bacterial α-diversity, evenness, and β-diversity. The figure shows the values of α-diversity (as bacterial OTUs richness assessed by ARISA), evenness (as Pielou index, *J*’), and bacterial β-diversity (as percentage of Bray-Curtis dissimilarity among sediment layers) for each layer along the vertical profile of the hadal sediments of the Izu-Bonin Trench. Small dots represent the values for each replicate, while the bigger dots and error bars report the average values and related SEs, respectively, for each sediment layer.

Molecular fingerprinting based on automated ribosomal intergenic spacer analysis (ARISA) indicated that the number of bacterial OTUs ranged from 36 to 107, with maximum values observed in the 120 cmbsf, and *J*’ evenness values increased from 0.79 ± 0.03 in surface sediments to 0.95 ± 0.02 in the deepest sediment layer at 150 cmbsf (Figure 5). These data suggested that the richness of bacterial OTUs (α-diversity) and their equitability (as *J*’, the Pielou evenness index) were high throughout the vertical sediment profile (Figure 5). Indeed, the values of bacterial OTUs richness reported here are comparable or higher than those reported so far for other deep-sea sediments at <6,000 m water depth using molecular fingerprinting techniques (range: 6–129 OTUs; Urakawa et al., 2001; Luna et al., 2004; Hewson and Fuhrman, 2007; Danovaro et al., 2009; Amaro et al., 2012). This suggests that the bacterial diversity in hadal sediments could be as high as in coastal and abyssal sediments, both in surface and subsurface sediment layers. Data provided here are not biased by possible contamination from extracellular DNA as the DNA extraction method used in the present study allows us to efficiently separate intracellular DNA from extracellular DNA (Corinaldesi et al., 2005, 2018; Dell’Anno et al., 2015; Danovaro et al., 2016). At the same time, since the cell extraction efficiency of the method applied to our samples has been reported to be ca. 20% (Corinaldesi et al., 2005; Luna et al., 2006), the actual amount of prokaryotic DNA in the trench sediments could have been underestimated. However, similarly low DNA extraction efficiency is typical also when using alternative commercial kits (Luna et al., 2006; Morono et al., 2014; Lever et al., 2015). To date, no procedure allows a 100% extraction efficiency of intracellular DNA from marine sediments (Luna et al., 2006; Morono et al., 2014; Lever et al., 2015). However, previous evidence indicates that different DNA extraction protocols applied to the same sediment sample can provide different DNA yields but similar estimates of bacterial diversity (Luna et al., 2006). Despite this evidence, additional studies based on 16S rRNA high-throughput sequencing are needed to confirm the bacterial diversity estimates obtained.

The bacterial turnover (β-)diversity among the sediment layers investigated in this study ranged from 31 to 90% dissimilarity of the bacterial assemblage composition (Figure 5). These findings suggest that the bacterial α-and β-diversity remained high also down to 1.5 mbsf in spite of the steep reduction in oxygen concentration and change in geochemical features of the sediments.

**Figure 6:**
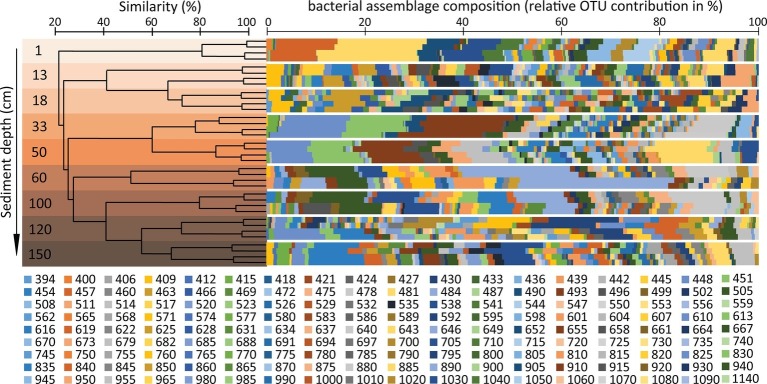
Analysis of similarity of the bacterial diversity in the different sediment layers of the Izu-Bonin Trench. The plot shows the clustering of the different samples analyzed in the present study, based on their similarity in bacterial assemblage composition as assessed through OTUs profiles identified by ARISA. The bacterial OTU ID indicates the base-pair lengths (see methods section) of the ARISA fragments. The multicolour bars show the bacterial assemblage composition for each sediment layer, each analyzed in four independent ARISA replicates.

The segregation of different bacterial OTUs along the sediment depth profile was highlighted by the cluster analysis (Figure 6), leading to hypothesize that the changes in geochemical conditions down to the sediment profile could influence the taxonomic and functional composition of the associated bacterial assemblages, with bacterial taxa of different metabolic roles inhabiting the different sediment layers. Further investigations based on metagenomics and metatranscriptomics or direct analysis of the bacterial metabolic activities are needed to test such a possibility in future studies.

Compared with typical oligotrophic deep-sea sediments, in which oxygen and nitrates penetrate deeper in the sediments (Durbin and Teske, 2011), our results suggest a spatial “niche squeezing effect” in organic-rich hadal sediments. In fact, in a relatively thin sediment layer of 1.5 mbsf, the fast rates of oxygen and nitrate consumption determined a steep vertical succession of multiple aerobic, hypoxic, and anaerobic niches, which might promote the high diversification of bacterial taxa we observed.

Our evidences are consistent with the presence of specific bacterial lineages observed in the different layers along the sediment vertical profile in the Mariana and Kermadec Trenches (Nunoura et al., 2018; Peoples et al., 2019). Despite several studies have proved that fingerprinting techniques, and especially ARISA, provide results consistent with those obtained using massive sequencing approaches in the assessment of bacterial diversity patterns (Schauer et al., 2010; Schöttner et al., 2011; Ristova et al., 2015; Jessen et al., 2017; Lindström et al., 2018), the contribution of “rare” members of the benthic assemblages could be underestimated to favor the detection of the more abundant members (Bent et al., 2007; Jones et al., 2012). Despite these limitations, fingerprinting techniques are still largely utilized in ecological studies aimed at investigating patterns and drivers of bacterial diversity on a wide variety of environmental samples (Billard et al., 2015; Cram et al., 2015; Fonti et al., 2015; Fuhrman et al., 2015; Witt et al., 2017; Lindström et al., 2018; Hao et al., 2019; Molari et al., 2019; Niederberger et al., 2019). Therefore, results reported here can contribute to expand our knowledge about the patterns of bacterial diversity in hadal sediments and provide new cues for future investigations in such remote ecosystems.

### Early Diagenesis of Organic Matter in Surface and Subsurface Hadal Sediments

**Figure 7:**
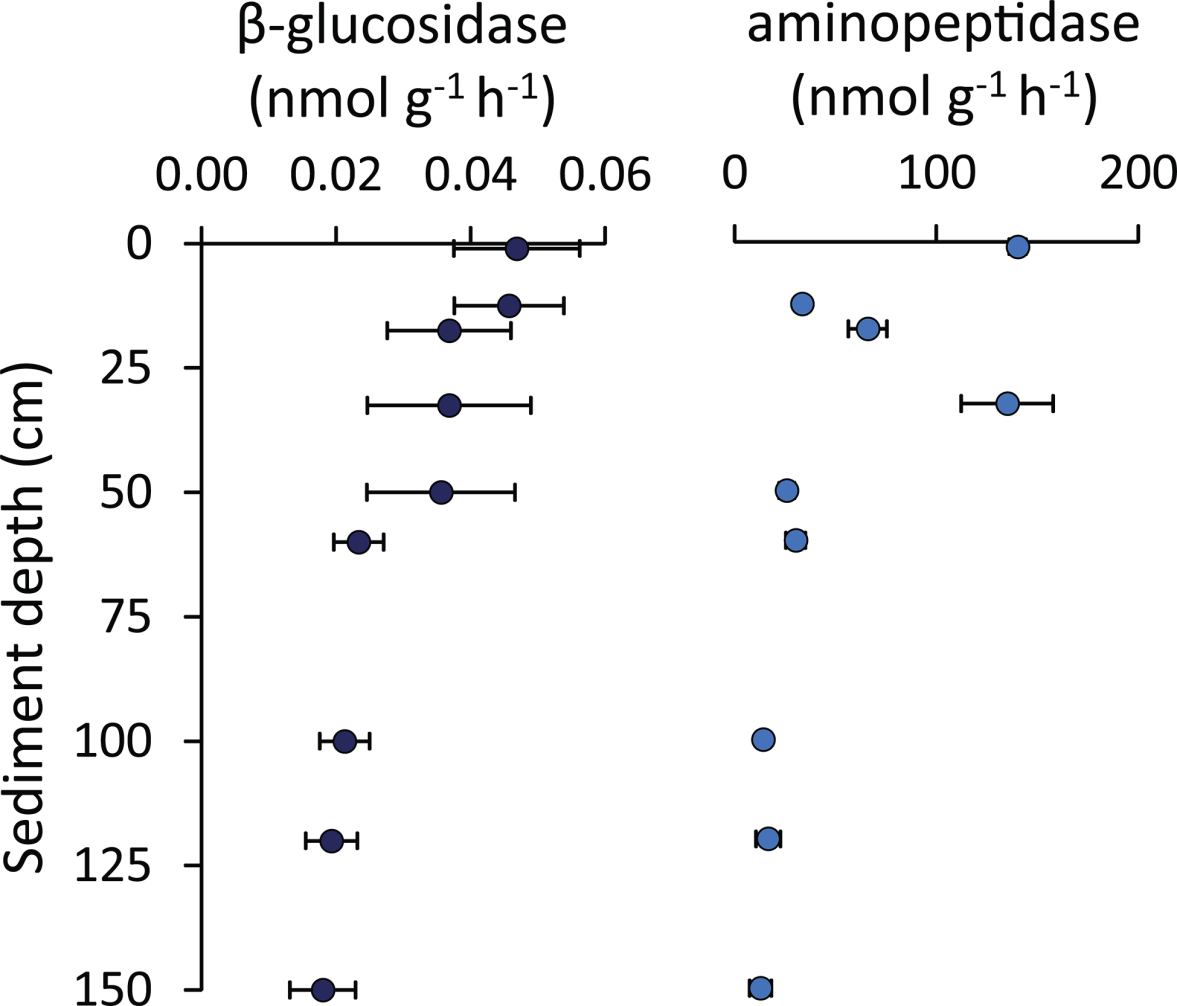
β-Glucosidase and aminopeptidase enzymatic activities. Reported are average values and SDs for the enzymatic activity rates determined for β-glucosidase and aminopeptidase along the vertical sediment profile.

The glucosidase and protease enzymatic activities generally decreased along the vertical sediment profile, but several subsurface layers (especially the 33-cm layer for aminopeptidase) displayed values similar to the rates observed in surface sediments (Figure 7). Notably, we found a peak of bioavailable organic resources (as biopolymeric C concentrations) in the 33-cmbsf layer, possibly contributing to explain the enhanced enzymatic activities observed at this sediment horizon (Figure 6).

The per-cell values of glucosidase and protease activity rates in subsurface sediments resulted in 0.001–0.003 and 0.9–2.7 fmol cell^−1^ h^−1^, respectively. These are similar or even higher than values observed in the top sediment layer (on average 0.001 and 1.8, respectively, for glucosidase and protease). Overall, when comparing surface and subsurface sediments, the comparable rates of viral production and the similar enzymatic activities rates per cell suggest that the shallow-subsurface sediments of the Izu-Bonin Trench host active bacteria that substantially contribute to the ongoing organic matter cycling processes.

**Figure 8:**
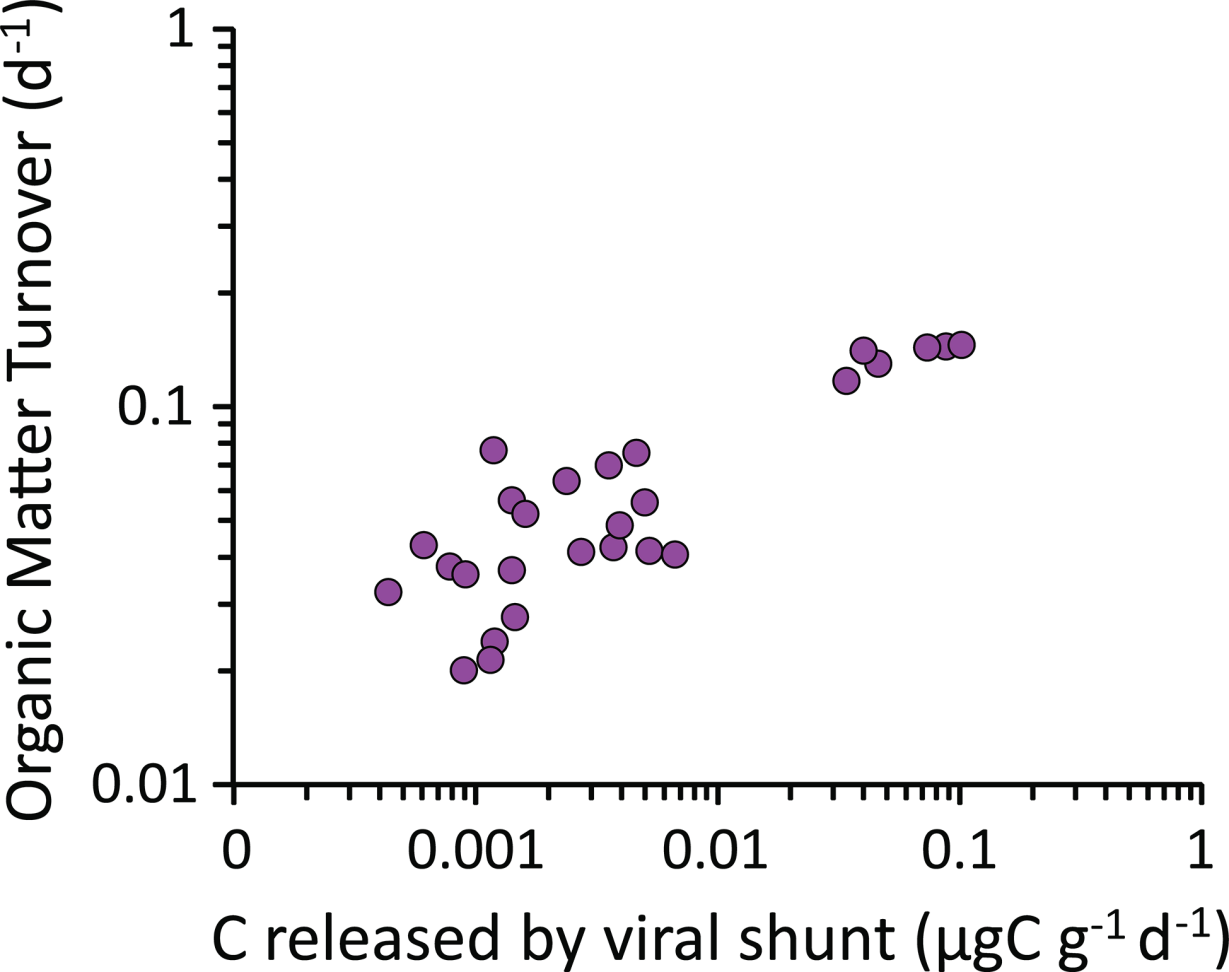
Relationship between the carbon released through the viral shunt and the organic matter turnover rates. The figure shows the positive correlation between the rates of carbon released following viral lysis of bacterial cells (i.e., viral shunt) and the organic matter turnover rates (as determined by enzymatic digestion of carbohydrates and proteins) in the hadal sediments analyzed in the present study.

In the present study, we found a positive and significant correlation between the rates of carbon release from viral lysis of bacteria and the organic matter turnover rates determined by enzymatic digestion of carbohydrates and proteins in the hadal sediments (*y* = 1.31; *x* = +4.46 × 10^−2^; *R*^2^ = 0.784; Figure 8). Viral lysis of bacteria in subsurface sediments, typically characterized by recalcitrant organic resources resistant to microbial degradation (Zonneveld et al., 2010), can supply highly bioavailabile compounds released from the burst of the infected cells (Middelboe et al., 2003; Poorvin et al., 2004; Weinbauer et al., 2011; Shelford et al., 2012; Weitz et al., 2015). Our results suggest that the virus-mediated release of such a labile material in subsurface sediments can trigger heterotrophic processes, accelerating the microbial-mediated organic matter early diagenesis. In terrestrial studies, this mechanism is known as organic matter “priming” (Chen et al., 2014; Perveen et al., 2014), and the role of viral lysis in enhancing this process has been investigated only recently in benthic marine ecosystems (Rastelli et al., 2017). These results lead to hypothesize that viruses can enhance microbial metabolism and accelerate organic matter cycling also in hadal subsurface sediments. At the same time, frequent earthquakes characterizing hadal trench systems can transfer large amounts of labile organic matter down to the trench bottom (Bao et al., 2018; Kioka et al., 2019). The sinking and accumulation of phytodetrital material along the trench axis can lead to concentrations of labile organic matter in the hadal trench sediments comparable with shallower habitats of high productivity (Danovaro et al., 2003; Jamieson, 2015, 2018; Wenzhöfer et al., 2016). These factors could also contribute to stimulate the rate of organic matter turnover in benthic hadal ecosystems (Glud et al., 2013; Wenzhöfer et al., 2016; Kioka et al., 2019).

### Drivers of the α- and β-Diversity and Functioning of the Benthic Bacterial Assemblages of the Izu-Bonin Trench

Despite bacterial assemblages play a major role in the ecology and biogeochemistry of subsurface sediments, information on the factors influencing their diversity, metabolic potential, and mortality rates is very limited (Nunoura et al., 2013, 2018; Kallmeyer et al., 2012; Orsi, 2018; Li et al., 2019: Peoples et al., 2019; Cui et al., 2019). A multivariate distance-based linear model (DISTLM) approach (Anderson et al., 2008) was used to obtain information on the drivers of the vertical patterns of bacterial diversity and functioning in the Izu-Bonin Trench sediments (Supplementary Table S1). Findings obtained here indicate that trophic resources (i.e., total organic carbon and biopolymeric carbon), porewater geochemistry of sediments (i.e., nitrate concentrations), and viral production rates contributed to explain a major portion of the variance of bacterial α-diversity (80%) along the vertical profile of the hadal sediments. Notably, nitrate concentration but not oxygen concentration was selected to explain the patterns of bacterial α-diversity, suggesting that the availability of nitrates along the hadal sediment profile and the differences in nitrogen-related metabolism might be particularly relevant in shaping the diversity of bacterial assemblages in this trench. At the same time, the variance of bacterial β-diversity was partially explained (overall, for 38%) by viral production, ammonium concentration, and trophic availability (i.e., total organic carbon). Other factors not investigated here could contribute to drive such bacterial β-diversity patterns, including sediment texture (Verma et al., 2017), episodic deposition in these tectonically active zones of allochtonous sediments of heterogeneous origin (Xu et al., 2018), and biotic drivers other than viruses (e.g., interactions of bacteria with archaea, fungi, and metazoa; Danovaro et al., 2016, 2017; Barone et al., 2018).

Overall, these results suggest that, in benthic hadal ecosystems, the patterns in the availability of trophic resources (in terms of quantity and quality of the organic matter) and geochemical features (mainly, nitrate and ammonium concentrations) have a key role in increasing the heterogeneity of niches available for different bacterial taxa along the vertical sediment profile. On the other hand, viral production is crucial in keeping high turnover diversity of bacterial taxa also in subsurficial sediments. In addition, since bacterial assemblage *J*’ evenness remained high down to 1.5 mbsf in the hadal sediments, we can argue that viruses, through the “killing-the-winner” strategy (Suttle, 2007), contributed to support high levels of bacterial diversity by avoiding dominance of specific bacterial OTUs. At the same time, it can be hypothesized that frequent earthquakes in these tectonically active zones (Bao et al., 2018; Kioka et al., 2019) and focusing of sedimentary material down to the trench bottom (Jamieson, 2015, 2018; Wenzhöfer et al., 2016) possibly contribute to increase benthic microbial diversity through the supply of cells attached to the deposited material (Boeuf et al., 2019). Even if the peculiar features of the hadal environment such as the exceptionally high hydrostatic pressure are thought to limit the immigration and adaptation of extrinsic microbes (Nunoura et al., 2018; Peoples et al., 2019), this aspect deserves further investigations in future studies on microbial assemblages in benthic hadal ecosystems.

Considering bacterial assemblage functioning (in terms of their potential in organic matter degradation processes), the main drivers were viral production rates, nitrate, oxygen, and total organic carbon concentrations (Supplementary Table S1). These results support the hypothesis that viral infections in subsurface hadal sediments, by accelerating the turnover of the most labile fraction of the biopolymeric organic carbon (proteins and carbohydrates), can promote organic matter priming processes, in turn enhancing organic matter cycling in benthic hadal ecosystems. At the same time, current evidences suggest that frequent earthquakes in these tectonically active zones can move large amounts of fresh and labile organic matter into trench bottom (Jamieson, 2015, 2018; Bao et al., 2018; Kioka et al., 2019), which could contribute to further enhance organic matter cycling.

## Conclusions

We show here high levels of α- and β-diversity of the bacterial assemblages along the vertical profile down to 1.55 mbsf in one of the deepest points of the largest hadal habitat on Earth: the Izu-Bonin Trench (Northwest Pacific Ocean). Viral infection and lysis processes, coupled with the quantity and quality of the sedimentary organic resources and of electron acceptors/donors available for microbial metabolism, were found to be the major drivers of the bacterial diversity patterns. We also found that larger bacterial standing stocks supported higher viral abundances and production rates and that the release of organic carbon through viral lysis of the infected bacterial hosts potentially promoted organic matter priming processes, in turn accelerating organic carbon cycling and nutrients regeneration in surface and subsurface hadal sediments.

Overall, our findings reveal that the changes in geochemical and trophic conditions at the hadal seafloor of the Izu-Bonin Trench can promote the creation of different niches along the sediment vertical profile. At the same time, viruses contribute to keep high richness and turnover of bacterial taxa and to enhance the organic matter cycling in subsurface hadal sediments, thus profoundly influencing the overall functioning of benthic hadal ecosystems.

## Data Availability Statement

The raw data supporting the conclusions of this manuscript will be made available by the authors, without undue reservation, to any qualified researcher.

## Author Contributions

RD, ER, CC, and AD’A conceived the study. ER, ML, TN, and HN provided samples for the study. ER, ML, MN, and MT performed laboratory analyses. ER, MT, AD’A, CC, and RD contributed to data elaboration. All authors contributed to data interpretation and discussion. TN and HN organized the oceanographic cruise and samples collection. ER, CC, and RD wrote the manuscript and all the authors contributed to the discussion and its finalization.

### Conflict of Interest

The authors declare that the research was conducted in the absence of any commercial or financial relationships that could be construed as a potential conflict of interest.
