# Supplementary material for: Drivers of Bacterial α- and β-Diversity Patterns and Functioning in Subsurface Hadal Sediments
Source: Front Microbiol. 2019 Nov 14;10:2609. doi: 10.3389/fmicb.2019.02609 (PMC6868121; doi:10.3389/fmicb.2019.02609)
Supplement: Supplementary file 1 [file Data_Sheet_1.docx]

**Supplementary Information**

**Drivers of bacterial α- and β-diversity patterns and functioning in subsurface hadal sediments**

Eugenio Rastelli, Cinzia Corinaldesi, Antonio Dell’Anno, Michael Tangherlini, Marco Lo Martire, Hidetaka Nomaki, Takuro Nunoura, Roberto Danovaro

**This file includes:**

Supplementary Figure 1 and 2

Supplementary Table 1


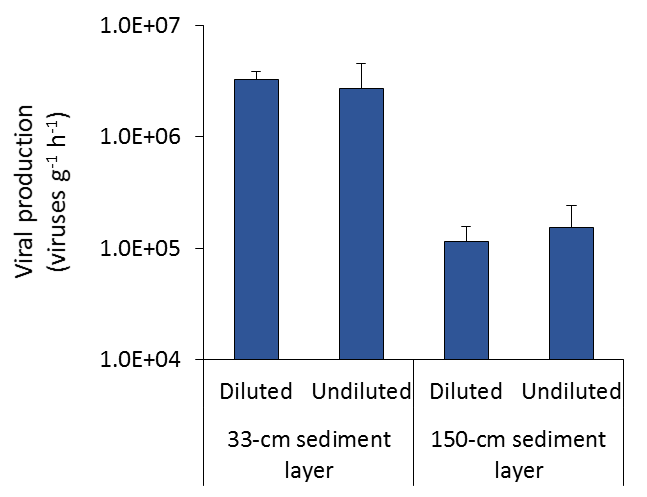


**Supplementary Figure 1. Comparison of viral production rates in diluted and undiluted sediments.** Reported are the viral production rates obtained from sediments incubated in parallel following the standard dilution approach (Dell’Anno et al., 2009) or as undiluted sediments (with no homogenization), to check for eventual effects of dilution and oxygen exposure of sediments.


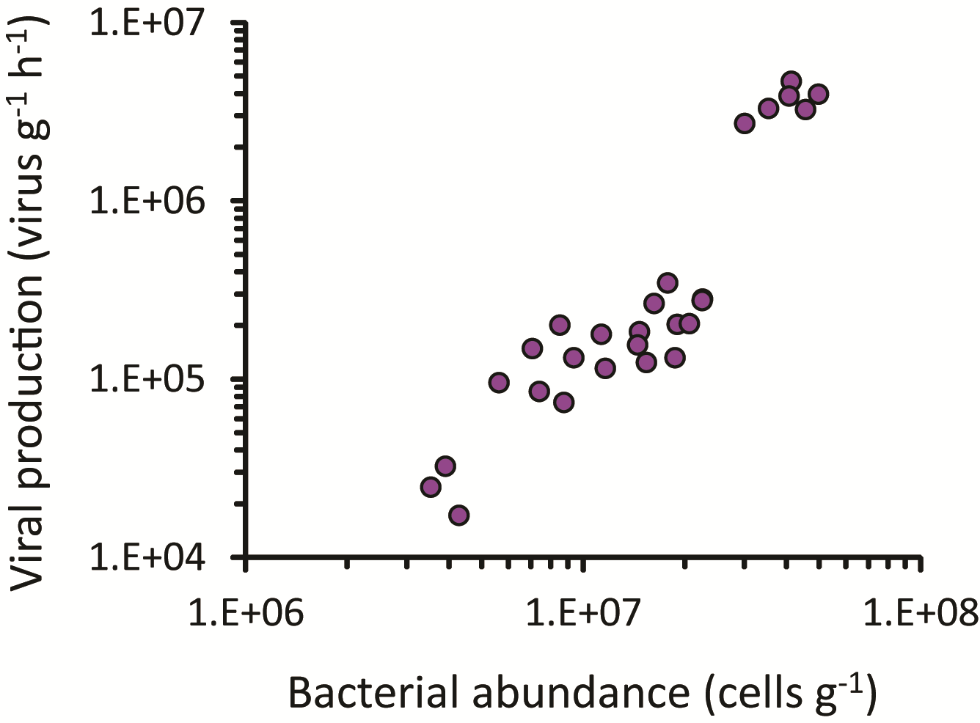


**Supplementary Figure 2. Relationship between bacterial abundance and viral production rates.** The figure shows the positive correlation between the values of bacterial abundance and of viral production rates in the hadal sediments analysed in the present study.

**Supplementary Table 1.** Environmental and biotic drivers of bacterial diversity and functioning in the hadal sediments of the Izu-Bonin Trench, based on DISTLM analysis outputs. SS: Sum of square. P-F: pseudo-F. Prop %: percentage of variance explained by each variable. VP, viral production; NH_4_^+,^ ammonium concentration; NO_3_^-^, nitrate concentration; P:C, protein-to-carbohydrate ratio; BPC, Biopolymeric carbon concentration; O_2,_ oxygen concentration; TOC; total organic carbon concentration. The cumulative percentage of variance is also reported. Only variables significantly contributing in explaining the observed variance are reported. P values are expressed as * (statistical significance at P<0.05), ** (P<0.01) and *** (P<0.001).

|  | **Variable** | **SS** | **P-F** | **P** | **cumulative prop (%)** |
| --- | --- | --- | --- | --- | --- |
| **α-diversity** | TOC | 1E+03 | 1E+01 | ** | 29.7 |
|  | VP | 7E+02 | 1E+01 | ** | 50.0 |
|  | BPC | 9E+02 | 3E+01 | *** | 77.1 |
|  | NO_3_^-^ | 1E+02 | 4E+00 | * | 80.0 |
| **β-diversity** | VP | 6E+01 | 4E+00 | * | 13.7 |
|  | NH_4_^+^ | 6E+01 | 4E+00 | * | 26.2 |
|  | TOC | 5E+01 | 4E+00 | * | 37.6 |
| **Organic matter turnover** | VP | 4E-02 | 169.35 | *** | 87.1 |
|  | NO_3_^-^ | 1E-03 | 6.6394 | * | 92.0 |
|  | O_2_ | 1E-03 | 9.4715 | ** | 94.6 |
|  | TOC | 7E-04 | 6.9519 | * | 96.0 |
